# Protective effects of *Lactiplantibacillus plantarum* YC225 Mixture Are associated with Gut Microbiota in Vulvovaginal Candidiasis

**DOI:** 10.4014/jmb.2601.01030

**Published:** 2026-04-27

**Authors:** Hyun Ju Kim, Dong-Gu Kim, Se-Woong Go, Jung-Hwang Park, Jong-Sik Jin, Hoonsung Choi, Ji-Hye Ahn, Iibum Park, Eui-Seong Park

**Affiliations:** 1 Kimchi Functionality Research Group, World Institute of Kimchi, Gwangju 61755, Republic of Korea; 2 Departments of Korean Medicine, Dong-eui University, Busan 47340, Republic of Korea; 3 Department of Pharmacy, Jeonbuk National University, Jeonju-si 54896, Republic of Korea; 4 Department of Oriental Medicine Resources, Jeonbuk National University, Iksan-si 54596, Republic of Korea; 5 Department of Korean Pharmacy, Woosuk University, Jeonbuk 55338, Republic of Korea; 6 Yuhan care Co., Ltd., Seoul 07335, Republic of Korea

**Keywords:** Vulvovaginal candidiasis, *Lactobacillus*, Inflammation, Vaginal tight junction, Gut microbiota, Probiotics

## Abstract

Vulvovaginal candidiasis (VVC) is a prevalent mucosal infection predominantly caused by *Candida albicans* (*C. albicans*). Vaginal lactobacilli contribute to colonization resistance and epithelial barrier integrity. However, therapeutic options remain limited and recurrence is common. In this study, we investigated the effects of a lactic acid bacteria mixture (LM; InoRexyne^®^) enriched with *Lactiplantibacillus plantarum* YC225 on antifungal activity and inflammation *in vitro* and in an estrogenized murine VVC model. In RAW264.7 macrophages and VK2/E6E7 vaginal epithelial cells, the LM cell-free supernatant inhibited *C. albicans* growth and reduced inflammatory readouts induced by lipopolysaccharide or *C. albicans*. *In vivo*, oral LM administration for 14 d attenuated VVC-associated histopathological damage, reduced vaginal IL-6 and nitric oxide levels, and decreased fungal burden compared with infected controls. LM also increased the expression of the tight junction marker gene *Tjp1* and modulated inflammatory gene expression in vaginal tissue (NanoString panel), including downregulation of selected pro-inflammatory pathways and upregulation of the anti-fibrotic regulator *Smad7*. 16S rRNA profiling of fecal samples showed LM-associated shifts in gut microbial composition, including increased abundance of *Lactobacillus*/*L. plantarum* and decreased abundance of several opportunistic taxa. Correlation analysis suggested associations between specific gut taxa and VVC parameters. Collectively, these findings indicate that oral LM may mitigate VVC-associated inflammation, epithelial damage, and fungal burden, and these effects are accompanied by changes in gut microbiota composition.

## Introduction

*Candida albicans* (*C. albicans*), the most prevalent human opportunistic pathogen, is the leading vaginal colonizer frequently observed from women with vulvovaginal candidiasis (VVC) worldwide [1]. Direct implications link *C. albicans* to vulvovaginal inflammation and VVC development, as evidenced by an increase in vaginal pH, lactobacilli depletion, pathogenic bacterial growth, biofilm formation, proinflammatory cytokines, epithelial barrier dysfunction, and alteration of the vaginal immune system during infection [2]. VVC breaks out about 75% of women in at least one event during their lives [2], and the cost for treating the disease is USD 368 million annually [3]. Azole antifungals, which are frequently employed to treat vaginal infections, inhibit ergosterol biosynthesis in fungal cell membranes, which increases membrane permeability and induces vaginal ecosystem dysbiosis, resulting in azole intolerance or resistance with overuse [4]. This dysbiosis is closely associated with increased inflammation, immune cell activation, and vaginal epithelial dysfunction observed in both VVC and bacterial vaginosis [5,6]. For this reason, significant interest has been paid to the selection and usage of probiotics, mainly lactobacilli, alone or in combination, to prevent or treat VVC by alleviating microbial dysbiosis through the production of metabolites [7].

VVC is increasingly viewed as an immunopathogenic inflammatory condition in which symptomatic disease is closely associated with a robust mucosal inflammatory response that does not necessarily correlate with effective fungal clearance [8–10]. In experimental models, hyphal formation and epithelial damage-associated signals can drive neutrophil recruitment and inflammatory mediators, contributing to tissue pathology and symptoms [9–11].

Over 50 lactobacilli species inhabit the vaginal ecosystem, mainly *L. crispatus*, *L. gasseri*, *L. inners*, and *L. jensenii*, and inhibit the adhesion, growth, proliferation, and colonization of *C. albicans* in the vaginal epithelium, predominantly by metabolites of gut bacteria [12, 13].

Probiotic lactobacilli have been explored to prevent or treat vaginal infections. However, clinical outcomes remain heterogeneous and are strain- and formulation-dependent, emphasizing the need to identify well-characterized strains with robust anti-*Candida* and immunomodulatory properties [14–16]. Increasing interest has focused on a potential gut–vagina axis in which oral probiotics may influence genital tract immunity or microbiota. However, evidence for consistent vaginal colonization following oral intake is mixed, and causal mechanisms remain unclear [17,18]. Therefore, studies that integrate host response, mucosal pathology, and gut microbial community changes may help clarify how oral lactobacilli formulations relate to vaginal outcomes.

Oral administration of UREX and Respecta^®^ probiotics, which have been authorized by the Korea Food & Drug Administration, maintains vaginal health by inhibiting pathogenic bacteria and yeast growth and adherence and increasing lactobacilli colonization, particularly in individuals with VVC and postmenopausal women [19–24]. Therefore, some pharmaceutical and food companies now exclusively sell products using the main ingredients of these probiotics, *L. reuteri* RC-14 and *L. rhamnosus* GR-1 from Chr. Hansen. Therefore, exploring lactobacilli species with universality or host specificity is a novel strategy for addressing vaginal microbiome dysbiosis in VVC [25].

*L. plantarum*, the key *lactobacillus* commonly used in fermenting Korean kimchi, exhibits anti-microbial, anti-inflammatory, anti-colitis, immunomodulatory, and anti-obesity properties [26]. In the present study, LPYC225 was derived from kimchi and revealed higher probiotic properties than the commercial probiotic *Lacticaseibacillus rhamnosus* GG. In our previous study, LPYC225 exhibited the highest anti-bacterial activity against *Gardnerella vaginalis* and anti-inflammatory effect in lipopolysaccharide-induced RAW 264.7 cells. The LPYC225 mixture partially ameliorated vaginal epithelial damage and modulated vaginal bacterial community by decreasing harmful bacteria in *G. vaginalis*-infected bacterial vaginosis, indicating that anti-bacterial effects of LPYC225 may be attributed to the production of H_2_O_2_ and short chain fatty acids in cell-free supernatants (CFS) [27].

In the present study, we screened lactic acid bacteria combinations and formulated a three-strain mixture enriched for *L. plantarum* YC225 (InoRexyne^®^) with the aim of maximizing anti-*Candida* and anti-inflammatory effects *in vitro*. We then tested whether the oral administration of this mixture mitigates pathology in an estrogenized murine VVC model and examined accompanying changes in vaginal inflammatory gene programs and gut microbiota composition. This integrated approach facilitated the evaluation of the therapeutic potential of the formulation and to explore associations between gut microbial shifts and VVC-related outcomes.

## Materials and Methods

### Preparation of Lactobacilli Strains and *C. albicans*

The lactobacilli strains *L. crispatus* [LCR01] and *L. fermentum* [LF05] originated from the vagina of healthy women, *L. plantarum* [LPYC225 and LPYC178] and *L. acidophilus* [LA02] (DSM21717) from fermented foods, *L. reuteri* from the vagina of healthy women, *L. rhamnosus* from healthy adult female urogenital track, and *C. albicans* (KCTC7122, 7729, 7678, and 7270) were provided by Yuhan Care Co., Ltd. (Republic of Korea) and stored at 4°C until use. To select the best probiotics, hemolytic activity, bile salt tolerance, acid tolerance, and adhesion ability were evaluated as part of a preliminary study. Among the strains, LPYC225 (KCTC19068P), originating from Korean kimchi, showed effects comparable to those of LGG (Table S1). LCR01, LF05, *L. reuteri*, and *L. rhamnosus* were purchased by Chr. Hansen (Denmark). For the experiment, the number of viable lactobacilli strains was counted in colony forming units (CFU). LM (InoRexyne^®^, Code No.: YC-NU-1106) was formulated with LPYC225, LCR01, and LF05 at a ratio of 7:2:1 based on final concentration (3.5 × 10^11^, 5.3 × 10^10^, and 5.7 × 10^10^ CFU/g). The 7:2:1 ratio was determined as the optimal formulation based on a comparison of data obtained from previous pilot tests and the combinations evaluated in this study (data not shown). UREX was purchased by Chr. Hansen (Hrsholm) and used as positive controls.

### Preparation of LAB and LAB Mixture CFS

Lactobacilli strains (LCR01, LF05, LPYC178, 225, LA02, *L. reuteri*, and *L. rhamnosus*) were anaerobically incubated on MRS agar at 37°C for 24 h. Each cultured lactobacilli strain was suspended in PBS (OD_660_, 1.0) and inoculated into 50 ml of MRS broth at a 1% concentration. After anaerobic incubation at 37°C for 24 h, each lactobacilli strain was centrifuged (4,000 rpm, 10 min). Each supernatant was filtered (0.22 μm), collected in 45 mL, and lyophilized. The lyophilized powder was dissolved in 4.5 ml of sterile distilled water to prepare the LAB CFS solution. The LAB CFS was stored at -20°C until use. The LAB mixture CFS solution was a mixture of LPYC225, LCR01, and LF05 CFS solutions, prepared immediately before use in experiments. Mixing ratios of LPYC225, LCR01 and LF05 CFS solutions were 4:5:1 (C2), 5:4:1 (C4), 6:3:1 (C6), and 7:2:1 (LM).

### Anti-*Candida* Activity of LAB and LAB Mixture CFS

A co-incubation assay using supernatants of selected *Lactobacillus* strains was performed as described by Hütt *et al*. [28], with some modifications. Briefly, *C. albicans* was seeded on potato dextrose agar and incubated in an aerobic chamber at 30°C for 24 h. The cultured *C. albicans* was suspended in PBS (OD_660_, 1.0) and inoculated into potato dextrose broth at a 1% concentration. LAB or LAB mixture CFS were treated into potato dextrose broth including *C. albicans* and incubated in an aerobic chamber at 30°C for 24 h. MRS broth without lactobacilli strain and LGG CFS were used as the control and positive control groups, respectively. Thereafter, potato dextrose broth containing *C. albicans* and CFS was serially diluted with PBS from 10^−5^ to 10^−8^ and inoculated on potato dextrose agar. CFU was assessed after incubation in an aerobic chamber at 30°C for 45 h. *C. albicans* colonies were counted to determine anti-*Candida* activity (%), which was calculated using the following formula: Anti-*Candida* activity = [1-(log CFU of test group/log CFU of control group)] × 100. The MIC and MFC of each LAB CFS were determined by CFU assay, and CFS was treated at a concentration of 17% (v/v) in the anti-*Candida* activity test.

### Cell Culture and LAB CFS Treatment of RAW264.7 Cells

For the cell study, lactobacilli strains were incubated in MRS broth in an anaerobic chamber at 37°C for 24 h. The murine RAW264.7 cells (ATCC TIB-71) were cultured in Dulbecco’s modified Eagle’s medium and seeded at 3 × 10^5^ cells/well for the nitric oxide (NO) assay and 3 × 10^6^ cells/dish for reverse transcription-quantitative PCR (qPCR). The supernatant of single LAB or LM was added at a 0.002% (v/v) 5 h before lipopolysaccharide treatment for 17 h for qPCR or 25 h for the NO assay [29]. The list of primers used is shown in Table S2.

### Cell Culture and LAB CFS Treatment of Vaginal Epithelial Cells

The human vaginal epithelial cell line VK2/E6E7 (ATCC CRL-2616) was cultured and seeded as previously described [30]. The cells were inoculated with *C. albicans* (2 × 10^4^ CFU/well) for 6 h, followed by treatment with the supernatant of single LAB or LM for 17 h. The supernatants were then gathered by centrifugation at 3,000 rpm for 5 min. Interleukin (IL)-β and IL-6 concentrations in the culture media were measured by ELISA kits (BD Biosciences, USA).

### Induction of *C. albicans*-Infected VVC Mice and LM Administration

Experiments using the VVC mice model were approved by the Institutional Animal Care and Use Committee of the World Institute of Kimchi (WIKIM IACUC 202236). An estrogen-dependent murine model of vulvovaginal candidiasis was established as previously described [31], with minor modifications. Briefly, mice were estrogenized to induce pseudoestrus and then inoculated intravaginally with *C. albicans* to achieve sustained vaginal colonization. To make VVC through *C. albicans* inoculation, mice were injected subcutaneously with β-estradiol-3-benzoate (0.5 mg/100 μl in olive oil) 3 and 4 d before and on the day of *C. albicans* inoculation. After 1 d, the mice were infected intravaginally with *C. albicans* (10^8^ CFU/20 μl) [31]. Mice were arbitrarily allocated to six groups (n = 10): NOR, *C. albicans*-infected control (CA), *C. albicans*-infected low LM (10^9^ CFU/mouse) (LML), *C. albicans*-infected middle LM (2 × 10^9^ CFU/mouse) (LMM), *C. albicans*-infected high LM (5 × 10^9^ CFU/mouse) (LMH), and *C. albicans*-infected positive control (10^9^ CFU/mouse) (UREX) groups. LM was orally administered each day for 14 d after *C. albicans* inoculation. LM dose was based on Kim *et al*. and Reid *et al*. [27, 32]. The mice were euthanized via CO_2_ asphyxiation at the end of the experiment to harvest the vaginal tissue and feces. The blood was collected from abdominal aorta and stored in a heparin tube. After centrifugation at 1,200 × *g*, for 10 min, at 4°C, plasma was aliquoted and stored at -80°C. The organs were stored at -80°C or in 10% buffered neutral formalin for histological analysis.

### Histology

To estimate the effect of LM on vaginal tissue damage in VVC mice, the histological changes and staining of *C. albicans* were analyzed via hematoxylin and eosin (H&E) and periodic acid-Schiff (PAS). Briefly, tissues were fixed in 10% formalin, embedded in paraffin, sectioned at 5 μm, stained with H&E and PAS, and observed under a microscope equipped with a camera (Axiocam 208 color) and an image analysis system (ZEN 3.3 blue edition) from Zeiss (Germany). The assessment of exfoliation and vaginal epithelial thickness was conducted as previously described [33].

### Pro-Inflammatory Cytokines and Myeloperoxidase Activity

To evaluate IL-6, NO levels and myeloperoxidase (MPO) activity, tissue was homogenized using RIPA lysis buffer (Sigma-Aldrich, USA) as previously described [33]. The supernatants were collected for the protein assay (#T9300A; TAKARA Bio INC, USA). The concentrations of IL-6 (#ab100712, Abcam, UK), MPO activity (#EK0943; Boster Bio Technology, USA), and NO (#G2930, Promega, USA) were measured using commercial ELISA and assay kits.

### Quantification of *C. albicans* in Washing Fluids

For quantification of *C. albicans* in vaginal lavage, mice were lightly anesthesia with CO_2_ and then vaginal lavage were gathered by flushing with 250 ul of sterile PBS. *C. albicans* (7.2 × 10^10^ CFU/ml), used as a standard, was cultured in YPD in a 30°C incubator for 24 h. qPCR was conducted by the QuantiTect SYBR^®^ Green PCR Kit (Qiagen), as previously described [34].

### RNA Extraction and qPCR in Vaginal Tissue

RNA was extracted from snap-frozen vaginal tissues by TRIzol reagent (Takara, Japan), and qPCR was performed by a SYBR Green qPCR kit (Promega), as previously described [30]. Briefly, the reaction conditions were based on a two-step cycling protocol, with pre-denaturation (95°C, 20 s) followed by 40 cycles at 95°C for 3 s and 60°C for 30 s. The product was amplified through real-time PCR and quantified using the ΔΔCt method by correcting the expression level of the house keeping gene *Gapdh*. The expression of *Tjp1* and *Foxa1* was compared to that in the NOR group. The list of primers used is shown in Table S3.

### NanoString Gene Expression Analysis

Briefly, 15 mg of vaginal tissue was extracted for total RNA. The RNA concentration and purity were measured by an Agilent 2100 Bioanalyzer (USA). All samples had an RNA integrity number > 8.5 (average = 9.35). mRNA expression was determined by the NanoString nCounter Analysis System (NanoString Technologies, USA) with a custom nCounter CodeSet of 37 genes involved in inflammation and lipid metabolism and three housekeeping genes. Raw data were normalized and processed as described by previous study [35]. Information of the target genes is shown in Table S4.

### Fecal Microbiome Analysis

Total genomic DNA was extracted from 200 μl of feces by a QIAamp DNA Stool Mini Kit (QIAGEN) and analysis of gut microbiota was carried out as previously described [36]. Linear discriminant analysis (LDA) effect size (LEfSe, v1.0) was used to analyze the differences in the relative abundance of bacterial taxa between groups. The parameters were set with the default *p* value, α = 0.05, and LDA score 3.0, and the results are depicted as bar or scatter plots.

### Statistical Analysis

Data were denoted as mean ± standard deviation. The cell study was independently repeated at least three times. Statistical analyses for *in vitro* and *in vivo* studies were carried out using SPSS 21.0 (USA) and GraphPad Prism 9.0.0 (USA), respectively. Variations observed across different groups were compared using one-way analysis of variance and Tukey’s multiple comparison test (*p* < 0.05). Correlation analysis and hierarchical clustering to the heat map of gut bacteria and VVC parameters was performed using Spearman’s rank correlation analysis and Ward's minimum variance method in JMP software (version 12.0.1; SAS Institute Inc., USA). The relationships between sample groups and the variable expression patterns were then visualized as a heat map with an accompanying dendrogram.

## Results

### Anti-Candida Activity and Anti-Inflammatory Effect of LAB or LAB Mixture

**Fig. 1:**
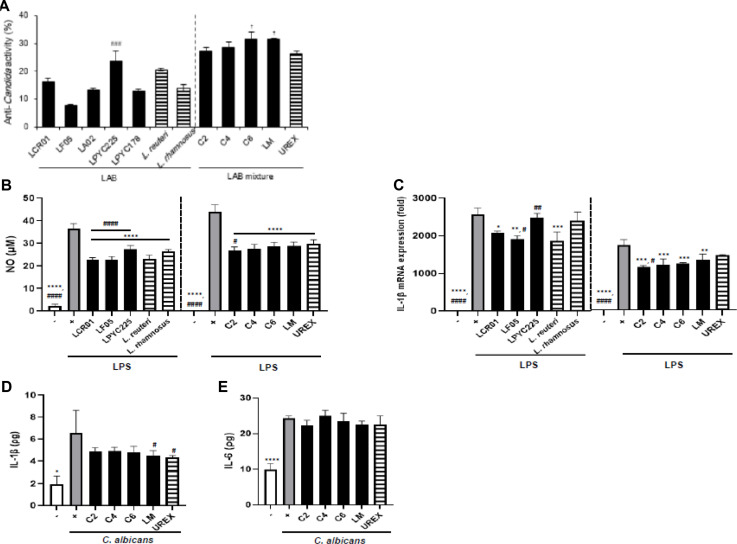
Anti-*Candida* activity and anti-inflammatory effect of LAB and LM. Anti-*Candida* activity, (**B**) NO in LAB and (**C**) LAB mixture, (**D**) *Il1b* mRNA expression in LAB and (**E**) LAB mixture in LPS-induced RAW264.7 cells. (**F**) IL-1β and (**G**) IL-6 production were measured by ELISA kit in *C. albicans*inoculated VK2/E6E7 cells. LCR: *L. crispatus*, LF: *L. fermentum*, LA: *L. acidophilus*, LP: *L. plantarum*. C2 (LCR01: LF05: LPYC225 = 5: 1: 4), C4 (LCR01: LF05: LPYC225 = 4: 1: 5), C6 (LCR01: LF05: LPYC225 = 3: 1: 6), LM (InoRexyne^®^-LCR01: LF05: LPYC225 = 2: 1: 7), UREX (*L. reuteri* : *L. rhamnosus*). Data were presented as the mean ± SD and analyzed by one-way ANOVA, followed by Dunnett’s multiple comparison post hoc test. *p < 0.05, **p < 0.01, ***p < 0.001, ****p < 0.0001 vs *L. reuteri*, *L. rhamnosus* or UREX. ^#^*p* < 0.05, ^##^*p* < 0.01, ^###^*p* < 0.001, ^####^*p* < 0.0001 vs LPS or *C. albicans*-treated control cells.

LAB and LAB mixture showed anti-*Candida* activity compared to *L. reuteri*, *L. rhamnosus*, and UREX (Fig. 1A). UREX is composed of *L. reuteri* and *L. rhamnosus* and commercially used for treating vaginitis worldwide. LPYC225 and LM showed higher anti-*Candida* activity than *L. reuteri*, *L. rhamnosus* against *C. albicans* strains KCTC7729, 7678, and 7270 (Table S5). Therefore, LPYC225, LCR01, and LF05 were combined at a ratio of 7:2:1 (LM) and selected for anti-*Candida* activity in the *C. albicans*-infected cell and animal model. Next, to clarify the anti-inflammatory effect of single LAB or LAB mixture, NO production and *Il1b* mRNA expression in lipopolysaccharide (LPS)-induced RAW264.7 cells were examined. Single LAB or LAB mixture significantly reduced NO levels compared to that in the LPS-treated control group. These effects were comparable to those of *L. reuteri*, *L. rhamnosus*, and UREX (Fig. 1B). However, the treatment of LCR01 and LF05 significantly inhibited *Il1b* mRNA expression compared to that in the LPS-treated control group, but not LPYC225. Moreover, the increase in *Il1b* mRNA expression in RAW264.7 macrophage cells induced by LPS was significantly inhibited following treatment with all of the LAB mixture (Fig. 1C). To determine the vaginal-specific anti-inflammatory effect of LM, *Il1b* and IL-6 levels in *C. albicans*-infected VK2/E6E7 cells were examined. *Il1b* and IL-6 levels increased following inoculation with *C. albicans*, but this effect was inhibited by application of all LAB mixtures, especially LM showed similar or higher effect than UREX (Fig. 1D and 1E).

### LM Relieves VVC by *C. albicans*-Infection

**Fig. 2:**
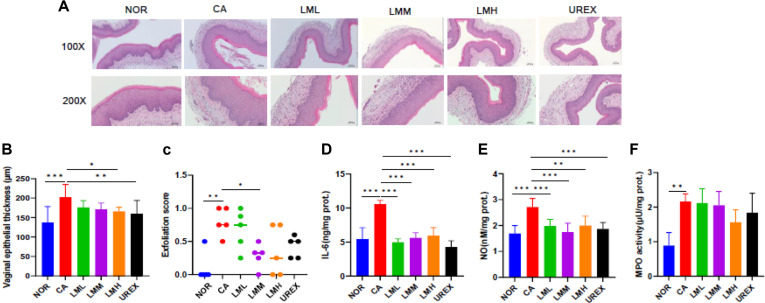
LM relieves vaginal epithelial in *C. albicans*-infected mice. (**A**) Representative H&E-staining (100×, 200×), (**B**) vaginal epithelial thickness and (**C**) exfoliation score (n = 5). (**D**) IL-6, (**E**) NO, (**F**) MPO activity in vaginal tissue. Data represent the mean ± SD (n = 10). Statistical significance was calculated using Prism 9. *p < 0.05, **p < 0.01, ***p < 0.001.

To figure out the effect of LM on VVC, mice were orally administered LM for 14 d. VVC mice showed marked increase of the vaginal epithelial cells and hyperkeratosis compared to the NOR group (Fig. 2A). However, administering LM significantly decreased vaginal epithelial thickness in the LMH group and the exfoliation score in the LMM group compared to the CA group (Fig. 2B and 2C). Next, we assessed the concentration of IL-6, NO, and MPO activity in vaginal tissue, which were significantly higher in the CA group than in the NOR group. Conversely, compared with the CA group, LM significantly reduced IL-6 and NO levels (Fig. 2D and 2E), but not MPO activity (Fig. 2F).

### LM inhibits *C. albicans* Growth and restores vaginal epithelial damage

**Fig. 3:**
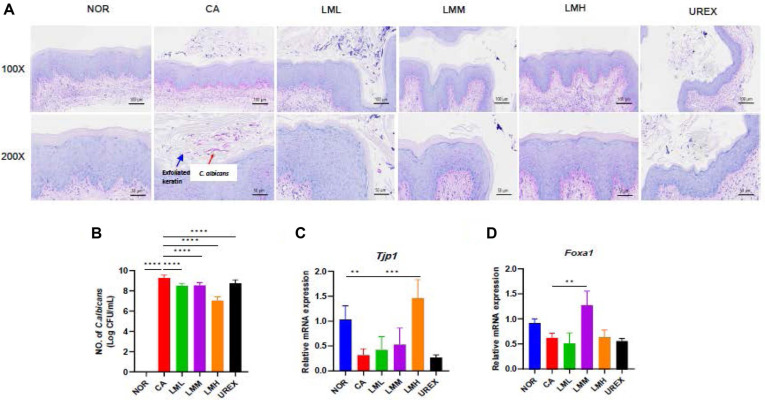
LM reduces burden of *C. albicans* and restores vaginal barrier function. (**A**) Histological evaluation of *C. albicans* by Periodic acid-Schiff (PAS) staining. Scale bar, 50, 100 μm. (**B**) Quantification of *C. albicans* in vaginal lavage were q-PCR. (**C**) mRNA expression of tight junction proteins, Zona occludens (*Tjp1*) and (**D**) Forkhead box A1/C2 (*Foxa1*) in vaginal tissue. Data represent the mean ± SD (n=5). Statistical significance was calculated using Prism 9. **p < 0.01, ***p < 0.001, ****p < 0.0001.

We investigated the vaginal *C. albicans* burden using PAS staining, which demonstrated marked numbers of *C. albicans* in vaginal mucosa of the CA group compared to those in the NOR group (Fig. 3A). The LM treatment groups showed a reduced number of *C. albicans* in the mucus and epithelium of the vaginal tissue, though this effect was more notable in the LML group than in the UREX group. Quantitative analysis of *C. albicans* in vaginal lavage was performed using qPCR. As expected, *C. albicans* was markedly higher in the CA group than in the NOR group (Fig. 3B). However, LM administration significantly inhibited *C. albicans* growth compared to that in the CA group. Furthermore, to investigate whether LM also exerts its beneficial effect on restoration of VVC condition by inhibiting the growth of *C. albicans* in the vagina when it is administered orally, we administered LMM via oral gavage for 2 weeks. The beneficial bacteria *L. plantarum* was significantly increased when compared to the NOR and CA groups (*p* < 0.05, Fig. S1A). Oral administration of LMM did not alter *L. fermentum* and tended to decrease the *C. albicans* growth in the vagina of *C. albicans*-infected mice, which was not a significant difference (Fig. S1B and C).

To assess tight junction-associated proteins, gene expression levels were examined. Expression of the tight junction protein zonula occludens (*Tjp1*) was lower in the CA group than that in the NOR group (Fig. 3C). Administering LM significantly upregulated *Tjp1* expression in the LMH group compared to that in the CA group, consistent with the vaginal epithelial damage observed following H&E staining. Interestingly, the expression of forkhead box A1 (*Foxa1*), a goblet cell marker, diminished in the CA group compared to that in the NOR group, whereas administering LMM significantly increased *Foxa1* expression (Fig. 3D), which may be associated with the reduction in vaginal epithelial exfoliation score and depleted mucus layer (Figs. 2C and 3A). These results suggest that the LPYC-enriched LAB mixture may play an essential role in maintaining a vaginal mucosal barrier against pathogens by increasing mucus secreted by vaginal goblet cells. Taken together, the results indicated that LM ameliorated vaginal epithelial damage and mucosal dysfunction in the VVC mouse model. To confirm the results from gene expression involved in tight junction-associated proteins, we performed ELISA assay in vaginal tissue of *C. albicans*-infected mice. A reduction of vaginal ZO-2 (TJP2) protein levels by *C. albicans* infection was significantly reversed by administration of LMM (Fig. S2A, *p* < 0.05), whereas the protein level of vaginal claudin-1 (Cldn1) was not changed by *C. albicans* infection (Fig. S2B).

### LM Reduces mRNA Expression of Inflammation-Related Factors in Vaginal Tissue

**Fig. 4:**
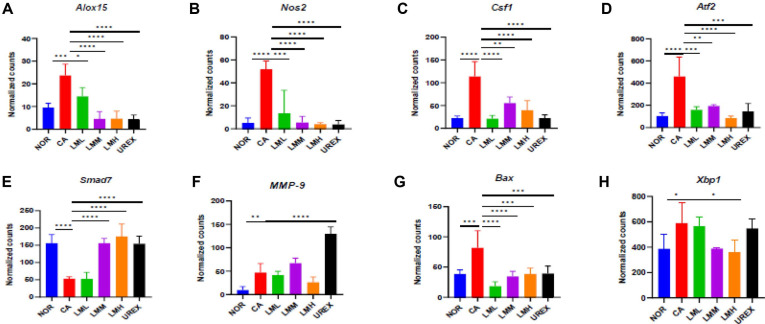
LM modulates genes expression involved in inflammation, fibrosis and apoptosis in vaginal tissue. (**A**) *Alox15*, (**B**) *Nos2*, (**C**) *Csf1*, (**D**) *Atf2*, (**E**) *Smad7*, (**F**) *MMP-9*, (**G**) *Bax*, (**H**) *Xbp1* were analyzed by NanoString gene expression. Data represent the mean ± SD (n = 5). Statistical significance was calculated using Prism 9. *p < 0.05, **p < 0.01, ***p < 0.001, ****p < 0.0001. *Alox15* arachidonate 15-lipoxygenase, *Nos2* nitric oxide synthase2, *Csf1* colony stimulating factor, *Atf* activating transcription factor, *Smad* SMAD family member, *MMP-9* matrix metalloprotein, *Bax* Bcl-2-associated X protein, *Xbp-1* X-box binding protein.

NanoString nCounter analysis system was applied to assess the expression of genes regarding inflammation. The results showed that the expression of genes involved in inflammation, fibrosis, and apoptosis genes were notably altered by *C. albicans* infection and that oral administration of LM downregulated genes involved in inflammation, such as *Alox15*, *Nos2*, *Csf1*, and *Atf2* (Fig. 4A–4D), consistent with the reduction of vaginal IL-6 and NO levels (Fig. 2D and 2E). Additionally, expression of *Smad7*, an antifibrotic factor, was reversed by administration of LMM, LMH, or UREX (Fig. 4E). Expression of *MMP-9*, a key factor in degrading the extracellular matrix, was not altered by administering LM, but administering UREX markedly increased its expression compared to that in the CA group (Fig. 4F). The increase in *Bax* and *Xbp1* expression following *C. albicans* infection was significantly decreased in the LM groups, especially the LMH group (Fig. 4G and 4H). The gene expression levels and heat map of the vaginal tissue are summarized in Table S6 and Fig. S2.

### LM Modulates Gut Microbiota of VVC Mice

**Fig. 5:**
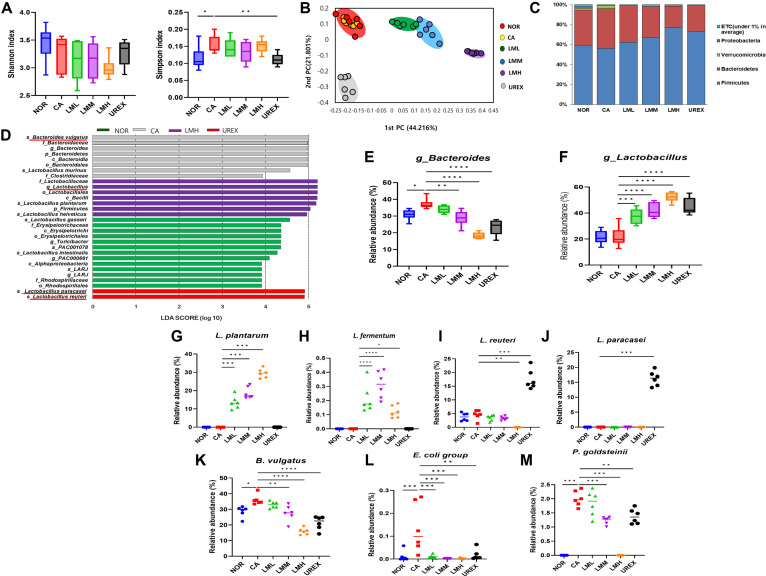
Administration of LM alters composition of fecal microbiota. (**A**) α-diversity as shown by Shannon (left) and Simpson (right) indices. (**B**) The β- diversity of gut microbiota was displayed by principal component analysis (PCoA) scatterplots. (**C**) The relative abundance of the main phyla. (**D**) Linear discriminant analysis (LDA) score of gut microbiota (with LDA score >3) at the each group. (**E**) *Bacteroides*, (**F**) *Lactobacillus*, (**G**) *L. plantarum*, (**H**) *L. fermentum*, (**I**) *L. reuteri*, (**J**) *L. paracasei*, (**K**) *P. vulgatus*, (**L**) *E. coli* group, (**M**) *P. goldsteinii*. Data represent the means ± SD (n = 6). Statistical significance was calculated using Prism 9. *p < 0.05, **p < 0.01, ***p < 0.001, ****p < 0.0001. Statistical significance was calculated using Wilkoxon rank-sum test. *p < 0.05, **p < 0.01 for α-diversity.

We performed 16S rRNA sequencing to analyze changes in gut microbiota composition in VVC mice. The α-diversity of bacteria was calculated by Shannon and Simpson indexes. The Simpson index was significantly higher in the CA group than that in the NOR group, indicating a VVC-induced decrease in microbial richness, which was reversed in the UREX group but not in the LM groups (Fig. 5A). β-Diversity revealed distinct clustering following probiotic administration (Fig. 5B). By analyzing the relative abundance of commensal microbiota at the phylum level, we found that administering LM increased the relative abundance of *Firmicutes*, whereas the relative abundance of *Bacteroides* decreased compared to that in the CA group (Fig. 5C). LEfSe analysis was performed to elucidate the characteristics of the top 30 taxa (LDA > 3), and the abundances of *P. vulgatus*, *Bacteroidetes*, *L. murinus*, and *Clostidiaceae* increased in the CA group (Fig. 5D). Furthermore, the increase in the relative abundance of *Bacteroides* at the genus level in the CA group was significantly reversed by LM administration (Fig. 5E). As expected, administering LM increased *Lactobacillus* (Fig. 5F) and *L. plantarum* in a dose-dependent manner (Fig. 5G and 5H). As expected, compared with the other groups, the UREX group showed a marked increase in the abundance of *L. reuteri* and *L. paracasei* (Fig. 5I and 5J). The relative abundance of *P. vulgatus*, *E. coli* group, and *Parabacteroides goldsteinii* at the species level was significantly higher in the CA group than in the NOR group, whereas a significant decrease was noted after administering LM (Fig. 5K–5M).

**Fig. 6:**
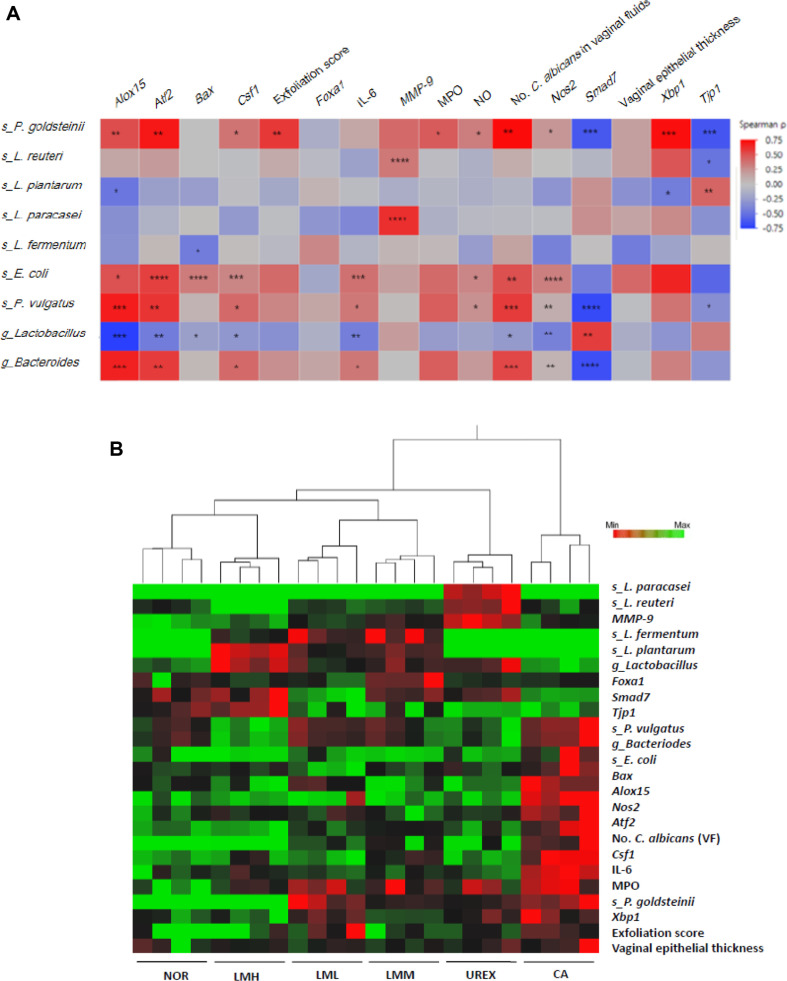
Heat map of the Spearman r correlation between the gut microbiota and VVC parameters (A) and hierarchical clustering analyses of VVC parameters across different groups in *C. albicans*-infected mice (B). Red and green denote highly and weakly expressed VVC parameters, respectively. *Alox15* arachidonate 15-lipoxygenase, *Atf* activating transcription factor, *Bax* Bcl-2-associated X protein, *Csf1* colony stimulating factor, e, *Foxa1* Forkhead box A1/C2, IL-6 *interleukin 6*, *MMP-9* matrix metalloprotein, NO nitric oxide, *Nos2* nitric oxide synthase2, *Smad* SMAD family member, *Xbp-1* X-box binding protein, *Tjp1* Zona occludens 1. *p < 0.05, **p < 0.01, ***p < 0.001, ****p < 0.001 following the Spearman correlation.

To ascertain the interaction between fecal microflora and VVC parameters, Spearman analysis was performed. *Lactobacillus*, *L. plantarum*, and *L. fermentum* abundance were negatively correlated with inflammatory factors and vaginal epithelial thickness, whereas they showed a significant positive correlation with *Tjp1* expression (Fig. 6A). *Bacteroides*, *P. goldsteinii*, *E. coli*, and *P. vulgatus* abundance were positively correlated with inflammatory factors and *C. albicans* burden, but negatively correlated with *Smad7* expression. Next, we performed hierarchically clustered heat map analysis to assess the overall patterns of VVC-related parameters, including vaginal epithelial thickness, exfoliation scores, inflammatory markers, and gut microbiota abundance across the groups. The resulting dendrogram separated the samples into two main clades. The CA group formed a distinct cluster, characterized by the upregulation of inflammatory and damage-associated variables and the downregulation of protective factors. Conversely, the NOR, all LM-treated, and UREX groups gathered together in the other main branch. Within this larger cluster, mice administered either the LM mixture or UREX exhibited physiological and microbial profiles that were clearly distinct from the pathological state of the CA group (Fig. 6B). These clustering results indicate that oral LM administration effectively attenuates *C. albicans*-induced microbial dysbiosis and tissue damage, shifting the overall profile toward that of the NOR group.

## Discussion

Our study delved into the protective effect of LM (InoRexyne^®^) against *C. albicans* in vaginal epithelial cell and VVC animal models. This study revealed that administering LM, which contains LPYC225, inhibited *C. albicans* growth and alleviated VVC, as evidenced by decreased inflammation, epithelial damage, *C. albicans* burden, increased epithelial barrier function, and modulation of the gut microbiota. The augment of pro-inflammatory cytokines, *Il1b* and IL-6, NO, and *Alox15*, *Nos2*, *Csf1*, and *Atf2* mRNA levels by *C. albicans* infection were attenuated by administering LM. Our data are consistent with other studies that observed that *C. albicans* evokes pro-inflammatory cytokine expression by activating NF-κB and the MAPK/AP-1 pathway, which is prevented by *L. plantarum* and *L. fermentum* in HeLa cells and vaginal epithelial VK2 cells [37, 38]. In the present study, anti-*Candida* activity of LPYC225, C6, and LM presented higher than *L. reuteri* and *L. rhamnosus*, and UREX, suggesting that anti-fungal effect of LM against *C. albicans* may be derived from LPYC225.

Of the three strains used in this experiment, we observed an increased abundance of LPYC225 in mouse vaginal lavage samples (Fig. S1). Furthermore, the increase in inflammatory cytokines was reduced by the single LAB and LAB mixture in lipopolysaccharide-induced RAW264.7 and VK2 cells, which was comparable to the results from UREX. Additionally, *L. crispatus* abrogates *C. albicans* growth and adherence by mediating the toll-like receptor 2/4 pathway, which is related to its anti-inflammatory effects [39]. Also, *L. crispatus* and *L. plantarum* strains from healthy human vaginas exhibit the best biofilm production under high glucose consumption conditions, which is attributed to their anti-*Candida* activity [40].

In our previous study, production of H_2_O_2_ from all of the single LAB in CFSs strongly suggests that anti-*Candida* activity is partially attributed to H_2_O_2_ production [27]. In this human study, LM supplementation at 2 × 10^9^ CFU/d was linked to improvements in vaginal microbiological and biochemical parameters, including a lower Nugent score and decreased elevated pH (data not shown). Given the largely immunopathology-driven nature of VVC, LM may primarily attenuate host inflammatory programs, and LPYC225 also exhibited vaginal adherence in mice. Thus, the observed reduction in fungal burden could represent a concomitant (secondary) outcome. Moreover, enhanced H_2_O_2_ production by LPYC225, in concert with biofilm formation documented in a prior study [40], may contribute to the eventual inhibition of *C. albicans* growth.

VVC pathogenesis is closely associated with the upregulation of pathways involved in inflammation, oxidative stress, fibrosis, and apoptosis [41, 42]. In our study, *C. albicans*-infected mice showed marked increases in the expression of inflammatory factors in vaginal tissue compared to normal mice, which is reversed by the administration of LM. Notably, the inflammatory factor IL-6 stimulates fibrosis through the TGF-β/SMAD pathway, and SMAD7, an anti-fibrotic factor, plays a protective role against inflammatory diseases and fibrosis [43]. In accordance to the protective role of SMAD7, the lack of *Smad7* expression likely boosted vaginal inflammation and fibrosis and induced apoptosis-related gene expression in our VVC mice, whereas administering LM increased *Smad7* expression. The mucosal epithelium acts as a physical barrier against microbial invasion [44]. Proteins produced by *C. albicans* hyphae disrupt vaginal barrier function and induce apoptosis, resulting in damage to epithelial cells [45]. In contrast, lactic acid produced by vaginal lactobacilli spp. enhances epithelial barrier function by upregulating the expression of genes encoding tight junction proteins, as observed in ectocervical epithelial cells and cohort studies [30]. In the present study, *C. albicans* triggered vaginal epithelial damage by increasing pro-inflammatory cytokines, inducing hyperkeratosis, and decreasing expression of the tight junction protein *Tjp1* and protein levels of Tjp2 (OZ-2) which was reversed by administering LM in VVC mice. Berard *et al*. [6] reported that vaginal epithelial barrier disruption and inflammation are associated with the activation of the microbiome-mechanistic target of rapamycin (mTOR) axis through imidazole propionate produced by the gut microbiota. Collectively, these results indicate that vaginal epithelial damage is associated with increased inflammation, fibrosis, and apoptosis and that LM has a protective effect against VVC. A more comprehensive investigation of the signaling pathway relevant to the vaginal epithelial dysfunction of VVC should be explored in future studies.

The yeast-to-hyphae transition is generally considered a key determinant of the virulence of *C. albicans* [46], inducing invasion, epithelial damage, immune activation, and neutrophil attraction in vaginal epithelial cells and VVC by activating MAPK transcription factors [47]. *L. johnsonii* notably reduces *C. albicans* hyphae and pro-inflammatory cytokines in vaginal tissue, alleviating epithelial damage and restoring normal vaginal architecture in VVC rats [42]. A combination of *L. crispatus* and *L. fermentum* showed anti-*Candida* activity, inhibiting *C. albicans* growth and hyphae formation by decreasing the expression of hyphal-related genes [48]. Our study revealed a decrease of *C. albicans* burden in the vaginal mucosa and washing fluid in the LM group compared to in the CA group, suggesting that LM exerted inhibitory effects on *C. albicans* growth and adherence in the vaginal tissue of VVC mice, even though this work not conceived hyphae transformation of *C. albicans* in vaginal tissue or lavage of mice. In addition, the oral administration of LM led to a significant increase in *L. plantarum* in the vaginal lavage of VVC mice, indicating the increase of *L. plantarum* in the vagina of VVC mice as a main mechanisms revealed by this study. The findings also make a strong case for the preventive potential of probiotics containing *L. plantarum* in the VVC states. This raises the possibility that vaginal microbes and their metabolites may affect epithelial function. Moreover, given that vaginal epithelial damage has been associated with increased inflammation, fibrosis, and apoptosis, LM could potentially confer protective effects in VVC.

To date, *L. rhamnosus* GR-1 and *L. reuteri* RC-14 are the most attractive probiotics for maintaining vaginal microbiome. Augmented evidence indicates that *lactobacillus* anti-*Candida* mechanisms involve inhibiting *C. albicans* growth and biofilm formation [37, 39], inducting coaggregation [49], producing antimicrobial substances [50], and modulating the immune system [45]. These effects are attributed to gut bacterial metabolites, short-chain fatty acids, lactic acid, and H_2_O_2_ [46]. Despite knowing some anti-*Candida* mechanisms, the relationship between gut microbiota and lactobacilli is underexplored in VVC mice models, warranting investigations for potential modulation strategies regarding gut microbiota. Clinical studies suggest that *L. crispatus*-predominant vaginal microbiota in women show decreased levels of pro-inflammatory cytokines [51]. Although *P. vulgatus* has been extensively scrutinized as a candidate for inflammation and metabolic disorders, its effects are still controversial [52–56] and it is entangled with a higher incidence of diseases including ulcerative colitis, obesity, and insulin resistance [52–54]. Conversely, *P. vulgatus* is the key player in gut microbiota and has favorable effects on acute intestinal injury, obesity, lipid metabolic disorders, and inflammatory arthritis [56, 57]. In this study, administering LM significantly diminished the relative abundance of *P. vulgatus* in a dose-dependent manner, concurrently alleviating epithelial damage and inflammation by reducing *C. albicans* burden of VVC mice. *C. albicans* stimulates *P. vulgatus* growth, and prevalence of *P. vulgatus* in type 2 diabetes has a positive correlation with insulin resistance and IL-6 levels [57]. Furthermore, *C. albicans* promotes *E. coli* growth and enhances virulence factors in women with candidiasis, which are inhibited by metabolites of lactobacilli [58, 59]. Herein, we found that the similarly augmented abundance of *E. coli* in the gut microbiota by *C. albicans* infection was inhibited by LM administration. Collectively, our study demonstrates that LM significantly increased the abundance of *Lactobacillus* and reduced *Bacteroides*, *P. vulgatus*, *E. coli*, and *P. goldsteinii* abundance in the gut microbial composition of *C. albicans*-infected mice, indicating that LM ameliorates VVC by modulating the overgrowth of opportunistic bacteria. Also, the increase in abundance of *L. plantarum* and the decrease in abundance of *P. vulgatus*, *E. coli*, and *P. goldsteinii* by LM was positively correlated with VVC parameters. The effect of LM will be studied using a germ-free mouse model to explore the effects of gut microbiota dysbiosis in VVC models.

Key limitations of the study include the largely correlational nature of the gut microbiota findings (precluding causal inference), the lack of direct vaginal microbiome/metabolite measurements, barrier-related conclusions being based primarily on transcript-level data rather than protein localization or functional permeability assays, and the need to disentangle strain-specific antifungal activity from nonspecific effects of supernatant acidification and to directly assess *Candida* virulence features (e.g., hyphal transition/biofilm). Collectively, the results justify further mechanistic and clinical validation of the formulation as an adjunct strategy for VVC.

## Conclusion

Oral administration of the LM enriched with *L. plantarum* YC225 reduced VVC-associated inflammatory responses, epithelial pathology, and fungal burden in an estrogenized murine model. These effects were accompanied by increased *Tjp1* transcript levels and coordinated changes in vaginal inflammatory gene expression, as well as shifts in fecal microbiota composition that correlated with disease parameters. Although our results support LM as a potential adjunct approach for VVC management, additional work, particularly assessing vaginal microbiota dynamics, active metabolites, and mechanistic causality between gut microbiota and vaginal outcomes, is needed to substantiate the gut–vagina axis contribution.

## Supplemental Materials

Supplementary data for this paper are available on-line only at http://jmb.or.kr.
