# Supplementary material for: Protective effects of Lactiplantibacillus plantarum YC225 Mixture Are associated with Gut Microbiota in Vulvovaginal Candidiasis
Source: J Microbiol Biotechnol. 2026 Apr 27;36:e2601030. doi: 10.4014/jmb.2601.01030 (PMC13146493; doi:10.4014/jmb.2601.01030)
Supplement: Supplementary file 1 [file jmb-36-e2601030-supple.pdf]

J. Microbiol. Biotechnol. 2026. 36: e2601030
https://doi.org/10.4014/jmb.2601.01030

Supplementary Materials and Methods

Hemolytic activity
Hemolytic activity was evaluated by streaking the selected strains onto blood agar plates and incubating them at 37°C for 48 h. Hemolysis was assessed by examining the presence or absence of a clear zone surrounding the colonies.

Acid tolerance
Selected probiotic strains were cultured in MRS broth at 37°C for 24 h. The cultures were then adjusted to an optical density of 0.6 at 660 nm and inoculated at 5% (v/v) into MRS broth adjusted to either pH 2.0 or pH 7.0. The inoculated cultures were incubated at 37°C with shaking for 30 min. After incubation, viable cell counts were determined to evaluate acid tolerance, and the viable cell count at pH 7.0 was used as the control.

Bile salt tolerance
Selected probiotic strains were cultured in MRS broth at 37°C for 24 h. The cultures were then adjusted to an optical density of 0.6 at 660 nm and inoculated at 5% (v/v) into MRS broth containing either 1% or 0% bile. The inoculated cultures were incubated at 37°C with shaking for 3 h. After incubation, viable cell counts were measured to assess bile tolerance, and the viable cell count in the absence of bile (0%) was used as the control.

Intestinal adhesion assay
HT-29 cells were seeded into 24-well plates at a density of 2 × 10^5 cells/well and incubated for 24 h. The selected probiotic strains were then

added to the cells at a multiplicity of infection (MOI) of 10 and incubated for 45 min. After incubation, non-adherent bacteria were removed by washing the cells three times with PBS. The cells were then lysed with 1% Triton X-100 (Sigma), and the bacteria associated with or internalized into the cells were serially diluted and plated for viable cell counting. The adhesion rate of the selected strains to intestinal epithelial cells was calculated using the following formula.

Quantification of vaginal microbial burden by quantitative PCR

Vaginal lavage samples were collected from mice and centrifuged to obtain microbial pellets. Genomic DNA was extracted from the pellets using the HiGene™ Genomic DNA Prep Kit (Biofact, Korea) according to the manufacturer's instructions. Quantitative PCR (qPCR) was performed to determine the abundance of *L. plantarum*, *L. fermentum*, and *C. albicans* using species-specific primers. The primer sequences used in this study were as follows: *L. plantarum* forward 5′-CCCAAAGCGGTAAGGTTGTT-3′ and reverse 5′-CTTCACGCTGGGGTCAACTT-3′; *L. fermentum* forward 5′-GCACCTGATTGATTTTGGTCG-3′ and reverse 5′-GTATTAGCAATCTGTTTCCAAATG-3′; and *C. albicans* forward 5′-ATGTGGCACGGCTTCTGCTG-3′ and reverse 5′-TAGGCTGGCAGTATCGTCAGAGG-3′. qPCR reactions were carried out in a total volume of 20 μL using 2× Real-Time PCR Master Mix Low ROX (Biofact, Korea; Cat. No. DQ384-40H). Each reaction contained the master mix, forward and reverse primers, template DNA, and nuclease-free water. Amplification was performed using an AriaMx Real-Time PCR System (Agilent Technologies, USA) under the following conditions: initial denaturation at 95 °C for 5 min, followed by 39 cycles of denaturation at 95 °C for 10 s, annealing at 60 °C for 30 s, and extension at 72 °C for 20 s. For absolute quantification, standard curves were generated using reference strains of *L. plantarum*, *L. fermentum*, and *C. albicans*. Each strain was cultured and the microbial suspension was serially diluted in ten-fold increments. Genomic DNA was extracted from each dilution using the same extraction method, and qPCR was performed to obtain Ct values. Standard curves were constructed by plotting Ct values against the logarithm of colony-forming units. Ct values obtained from vaginal lavage samples were interpolated from the corresponding standard curves to calculate the microbial burden, and the results were expressed as fold change compared to the NOR group based on the analysis of △△Ct values.

Tight junction protein levels in vaginal tissues

Vaginal tissues, 50 mg were used and homogenized in RIPA buffer (Thermo Fisher Scientific, Seoul, South Korea) containing 1% protease

inhibitor cocktail and 1% DTT. Supernatants were collected after centrifugation. Mouse ZO-2 and Claudin-1 (Cldn1) ELISA kit were purchased from Abbkine (Atlanta, GA, USA) and FineTest (Wuhan, Hubei, China).

Supplementary data

Table S1. Probiotic effect of lactic acid bacteria (LAB).

Strain NO.	Identification	Hemolytic activity	Bile salt tolerance	Acid tolerance	Adhesion ability
LPYC-71	*L. plantarum*	-	98.50 ± 1.35	43.93 ± 0.37	78.33 ± 0.36
LPYC-114	*L. plantarum*	-	98.51 ± 2.63	44.91 ± 0.82	79.02 ± 0.80
LPYC-155	*L. plantarum*	NE	99.37 ± 0.24	46.54 ± 4.56	74.73 ± 1.23
LPYC-163	*L. plantarum*	NE	98.51 ± 2.51	44.11 ± 3.55	71.15 ± 0.85
LPYC-178	*L. plantarum*	-	97.71 ± 0.27	$63.08 \pm 1.16^{**}$	80.09 ± 0.50
LPYC-225	*L. plantarum*	-	103.14 ± 2.45	$65.27 \pm 5.42^{***}$	81.29 ± 0.90
LPYC-287	*L. plantarum*	NE	$93.28 \pm 1.22^{**}$	$63.90 \pm 6.34^{***}$	74.91 ± 0.13
LPYC-333	*L. plantarum*	-	98.35 ± 1.23	52.43 ± 6.41	76.43 ± 1.21
LPYC-350	*L. plantarum*	-	99.38 ± 0.27	53.93 ± 1.43	77.88 ± 0.47
LPYC-363	*L. plantarum*	NE	$94.33 \pm 3.46^{*}$	49.86 ± 4.93	72.79 ± 1.03
LGG	*L. rhamnosus*	NE	101.32 ± 2.82	42.42 ± 4.54	$86.78 \pm 0.99^{\#\#\#\#}$

NE: Not experiment.

$^{*}p<0.05$, $^{**}p<0.01$, $^{***}p<0.001$ vs LGG.

$^{\#\#\#\#}p<0.0001$ vs single LAB.

Table S2. List of primers used for RT-qPCR in RAW264.7 cells

List of genes	Forward	Reverse
Illb	5'-CCTCGTGCTGTCGGACCCAT-3'	5'-CAGGCTTGTGCTCTGCTTGTGA-3'
Gapdh	5'-TGTGTCCGTCGTGGATCTGA-3'	5'-TTGCTGTTGAAGTCGCAGGAG-3'

Il1b = Interleukin -1β

Table S3. List of primers used for qPCR in vaginal tissue

List of genes	Forward	Reverse
Gapdh	5'-ACCCAGAAGACTGTGGATGG-3'	5'-TTCTAGACGGCAGGTCAGGT-3'
Tjp1	5'-TAACTTGGGGAGGGAGGGTC-3'	5'-GGTAAGGCATTCCTGCTGGT-3'
Foxa1	5'-GAGAGAAAAAAUCAACAGC-3'	5'-GCUGUUGAUUUUUUCUCUC-3'

Tjp1 = Zona occludens; *Foxa1*= Forkhead box A

Table S4. Nanostring gene list

**Name**	**Accession no.**	**Target sequence**
Alox15	NM_009660.3	CAGCTGGATGAGGAGCTCAAGAAAGGCACTCTGTTTGAAGCGGATTTCTTCCTTCTGGATGGGATCAAGGCCAATGTCATCCTTTGTAGTCAGCAGTACC
Ppara	NM_011144.6	GACCTTGGAAAGGGAGTTTTGAGTCATGGGCTTTCGGGATAGTTGAGCATTCTGTTTGAACTCGCCTAAAACTTATGAAGAGTAGTCCCTTGCTGTGTGC
Pparg	NM_001127330.1	GAGCAAAGAGGTGGCCATCCGAATTTTTCAAGGGTGCCAGTTTCGATCCGTAGAAGCCGTGCAAGAGATCACAGAGTATGCCAAAAATATCCCTGGTTTC
Adipoq	NM_009605.4	GACCACAATGGACTCTATGCAGATAACGTCAACGACTCTACATTTACTGGCTTTCTTCTCTACCATGATACCAACTGACTGCAACTACCCATAGCCCATA
Lep	NM_008493.3	CCTATTGATGGGTCTGCCCAAGGCAAACCTAATTTTTGAGTGACTGGAAGGAAGGTTGGGATCTTCCAAACAAGAGTCTATGCAGGTAGCGCTCAAGCTT
Cd36	NM_007643.3	GGGACCATTGGTGATGAAAAAGCAGAAATGTTCAAAACACAAGTGACTGGGAAAATCAAGCTCCTTGGCATGGTAGAGATGGCCTTACTTGGGATTGGAG
Cptla	NM_013495.2	GCGAAGTGTCGGCAGACCTATTTTGCACGAGGAAAAAATAAGCAATCTCTGGATGCGGTAGAAAAGGCAGCATTCTTCGTGACGTTGGACGAATCGGAAC
Scd1	NM_009127.4	GGAGGCCTGTACGGGATCATACTGGTTCCCTCCTGCAAGCTCTACACCTGCCTCTTCGGGATTTTCTACTACATGACCAGCGCTCTGGGCATCACAGCCG
Srebf1	NM_011480.4	GGTTGCTGCCACCCCTAGTCTGGCTGGCCAATGGACTACTAGTGTTGGCCTGCTTGGCTCTTCTCTTTGTCTATGGGGAACCTGTGACTAGGCCACACTC
LPL	NM_008509.2	ACGCTGATTTTGTAGATGTCTTACACACATTTACCAGGGGGTCACCTGGTCGAAGTATTGGAATCCAGAAACCAGTAGGGCATGTTGACATTTATCCCAA
Bax	NM_007527.3	CATAAATTATGACATTTTCCTGGGATGAATGGGGGAAGGGGAAAGGCATTTTTCTTACTTTTGTAATTATTGGGAGGGGTGGGAATGGTGGCCTGGGGAG
Bcl2	NM_009743.4	GAGCAACCGGGAGCTGGTGGTCGACTTTCTCTCCTACAAGCTTTCCCAGAAAGGATACAGCTGGAGTCAGTTTAGTGATGTCGAAGAGAATAGGACTGAG
Atf2	NM_001284371.1	AGAGCAGCAGCTTCAAGATGCCGACAAAAAAGGAAAGTGTGGGTTCAGTCCTTAGAGAAGAAAGCAGAAGACTTGAGTTCACTAAATGGCCAGCTGCAGG
Creb1	NM_001037726.1	TTCTGTCTGGACAGTTCACCAGATTCTCCAGAAGGCTTTCAAACGGCTAAAGTTTGATCTTTGTCCTGCTGAGCTTGCTGGGAAGGAGATAGCATAAAAG
Crp	NM_007768.4	TTGTATTTCCCAAGGAGTCAGATACTTCCTATGTGTCTCTGGAAGCAGAGTCAAAGAAGCCACTGAACACCTTTACTGTGTGTCTCCATTTCTACACTGC
Csf1	NM_001113530.1	TCCAGCTGCTGGAGAAGATCAAGAACTTCTTTAATGAAACAAAGAATCTCCTTGAAAAGGACTGGAACATTTTTACCAAGAACTGCAACAACAGCTTTGC
Cxcl12	NM_021704.3	CTGAAAATCCTCAACACTCCAAACTGTGCCCTTCAGATTGTTGCACGGCTGAAGAACAACAACAGACAAGTGTGCATTGACCCGAAATTAAAGTGGATCC
Il10	NM_010548.2	GACAACATACTGCTAACCGACTCCTTAATGCAGGACTTTAAGGGTTACTTGGGTTGCCAAGCCTTATCGGAAATGATCCAGTTTTACCTGGTAGAAGTGA

Il17a	NM_010552.3	AGGGAGAGCTTCATCTGTGTCTCTGATGCTGTTGCTGCTGCTGAGCCTGGCGGCTACAGTGAAGGCAGCAGCGATCATCCCTCAAAGCTCAGCGTGTCCA
Il6	NM_031168.1	ATGTTCTCTGGGAAATCGTGGAAATGAGAAAAGAGTTGTGCAATGGCAATTCTGATTGTATGAACAACGATGATGCACTTGCAGAAAACAATCTGAAACT
Il22	NM_016971.1	AGAAGAATGTCAGAAGGCTGAAGGAGACAGTGAAAAAGCTTGGAGAGAGTGGAGAGATCAAGGCGATTGGGGAACTGGACCTGCTGTTTATGTCTCTGAG
Jun	NM_010591.2	CGCGACCAGAACGATGGACTTTTCGTTAACATTGACCAAGAACTGCATGGACCTAACATTCGATCTCATTCAGTATTAAAGGGGGGTGGGAGGGGTTACA
Keap1	NM_016679.4	TCTATTGTCTCTGCTTGCCATTGTACATTCTGCTCAGACAGGGCATCTTGCTTCTTGTGGGACACACAGTTGTCTGTCAGTTTCAGGGCATTAGAAGCCA
Mapk3	NM_011952.2	ACCTTAATTGCATCATTAACATGAAGGCCCGAAACTACCTGCAGTCTCTGCCCTCGAAAACCAAGGTGGCTTGGGCCAAGCTCTTTCCTAAATCTGACTC
MMP-9	NM_013599.2	GAGCCAGGCTCTCTACTGGGCGTTAGGGACAGAAATGTTGGTTCTTCCTTCAAGGATTGCTCAGAGATTCTCCGTGTCCTGTAAATCTGCTGAAACCAGA
Nfkb1	NM_008689.2	GTCTTACACTTAGCCATCATCCACCTCCACGCTCAGCTTGTGAGGGATCTGCTGGAAGTCACATCTGGTTTGATCTCTGATGACATCATCAACATGAGAA
Nfkbia	NM_010907.2	CTGCAGGCCACCAACTACAATGGCCACACGTGTCTGCACCTAGCCTCTATCCACGGCTACCTGGCCATCGTGGAGCACTTGGTGACTTTGGGTGCTGATG
Nos2	NM_010927.3	AGACTGGATTTGGCTGGTCCCTCCAGTGTCTGGGAGCATCACCCCTGTGTTCCACCAGGAGATGTTGAACTATGTCCTATCTCCATTCTACTACTACCAG
Nox1	NM_172203.1	CTCCAAACATGACAGTGATGTATGCAGCATTTACCAGTATTGCTGGCCTTACTGGAGTGATTGCCACTGTAGCTTTGGTTCTCATGGTAACGTCAGCTAT
Ptgs2	NM_011198.4	GACCTGGGTTCACCCGAGGACTGGGCCATGGAGTGGACTTAAATCACATTTATGGTGAAACTCTGGACAGACAACATAAACTGCGCCTTTTCAAGGATGG
Rac1	NM_009007.2	GCCAAAATACCTTCTGAACTAAGTTGCGTTGTGCTGAGAACACCTAAGCACTAAACTCTCTTGAGAGACTTCTGTTGCTAAGAAGACCGCAGCTTCTGGA
Rela	NM_009045.4	GGCACGAGGCTCCTTTTCTCAAGCTGATGTGCATCGGCAAGTGGCCATTGTGTTCCGGACTCCTCCGTACGCCGACCCCAGCCTCCAGGCTCCTGTTCGA
Smad7	NM_001042660.1	CAGCCTGCTAGAAGGCTGAACCAGAACCAATTGTTTTCATCCCTGTCTTACTGCCGCCTGTCACCCGCTGCCATTGTCGAGTCTGTCTTTTTTGGCCATC
Stat3	NM_213659.2	AGCTTAAAATTAAAGTGTGCATTGATAAAGACTCTGGGGATGTTGCTGCCCTCAGAGGGTCTCGGAAATTTAACATTCTGGGCACGAACACAAAAGTGAT
Tgfb2	NM_009367.4	TGGTGAAGGCAGAGTTCAGGGTCTTCCGCTTGCAAAACCCCAAAGCCAGAGTGGCCGAGCAGCGGATTGAACTGTATCAGATCCTTAAATCCAAAGACTT
Tnf	NM_013693.2	TGGATCTCAAAGACAACCAACTAGTGGTGCCAGCCGATGGGTTGTACCTTGTCTACTCCCAGGTTCTCTTCAAGGGACAAGGCTGCCCCGACTACGTGCT
Xbp1	NM_013842.2	AGAACCACAAACTCCAGCTAGAAAATCAGCTTTTACGGGAGAAAACTCACGGCCTTGTGGTTGAGAACCAGGAGTTAAGAACACGCTTGGGAATGGACAC
Gapdh	NM_008084.1	ATGTGTCCGTCGTGGATCTGACGTGCCGCCTGGAGAAACCTGCCAAGTATGATGACATCAAGAAGGTGGTGAAGCAGGCATCTGAGGGCCCACTGAAGGG
HPRT1	NM_013556.2	TGCTGAGGCGGCGAGGGAGAGCGTTGGGCTTACCTCACTGCTTTCCGGAGCGGTAGCACCTCCTCCGCCGGCTTCCTCCTCAGACCGCTTTTTGCCGCGA
PLR1B	NM_009086.2	TGCCTTTCACTGAGAGTGGCATGATGCCGGACATTCTGTTTAATCCTCACGGGTTTCCCTCCCGTATGACCATAGGTATGTTAATCGAGAGCATGGCTGG

Table S5. Anti-*Candida* activity of LAB against *C. albicans*.

LAB	Anti-*Candida* activity (%)		
	KCTC7729	KCTC7678	KCTC7270
LCR01	23.2 ± 4.6	31.7 ± 10.8	23.4 ± 3.2
LF05	6.6 ± 4.7	15.6 ± 4.7	10.3 ± 7.8
LA02	32.3 ± 11.5	25.3 ± 3.2	28.0 ± 4.6
LP YC-225	56.8 ± 12.8$^{†††,\#\#\#}$	37.1 ± 4.6	42.0 ± 6.3$^{\#\#}$
LP YC-178	14.4 ± 3.4	28.2 ± 5.1	14.0 ± 6.1
L. reuteri	27.5 ± 2.3	33.0 ± 7.0	32.7 ± 3.4
L. rhamnosus	16.5 ± 2.7	33.6 ± 7.0	17.5 ± 6.1

LCR: *L. crispatus,* LF: *L. fermentum,* LP: *L. plantarum*, LA: *L. acidophilus.*

Data were presented with independent three repeated experiment. Statistical significance was determined using one-way ANOVA, followed by Dunnett's multiple comparison post hoc test. $^{†}p<0.05$, $^{†††}p<0.001$ vs *L. reuteri*, $^{\#}p<0.05$, $^{\#\#}p<0.01$, $^{\#\#\#}p<0.001$ vs *L. rhamnosus*.

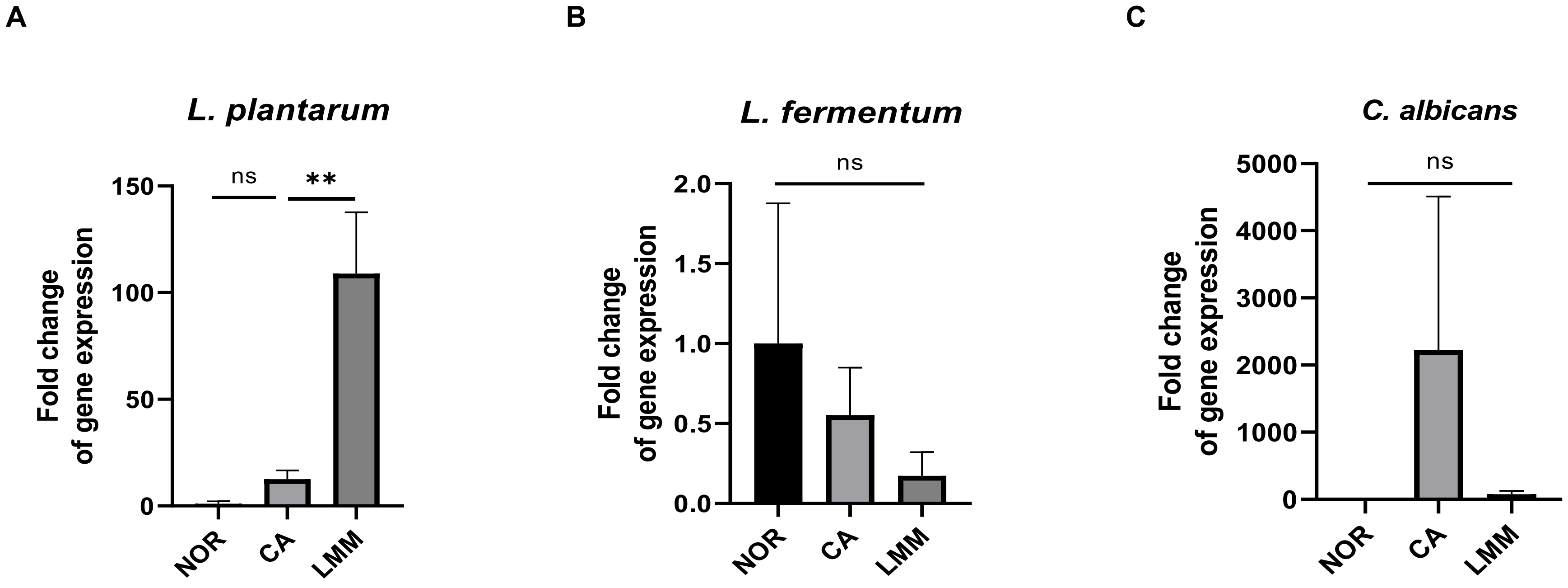

Fig. S1. **qRT-PCR of *L. plantarum*, *L. fermentum*, and *C. albicans* in the vaginal lavage from *C. albicans*-infected mice.**

Data represent the mean ± SD (n=5).

Statistical significance was calculated using Prism 9. $^{*}p<0.05$.

NOR: Normal group

CA: *C. albicans* (1×10^8 CFU/20ul) infected group

LMM: *C. albicans*-infected middle LM (2×10^9 CFU) group

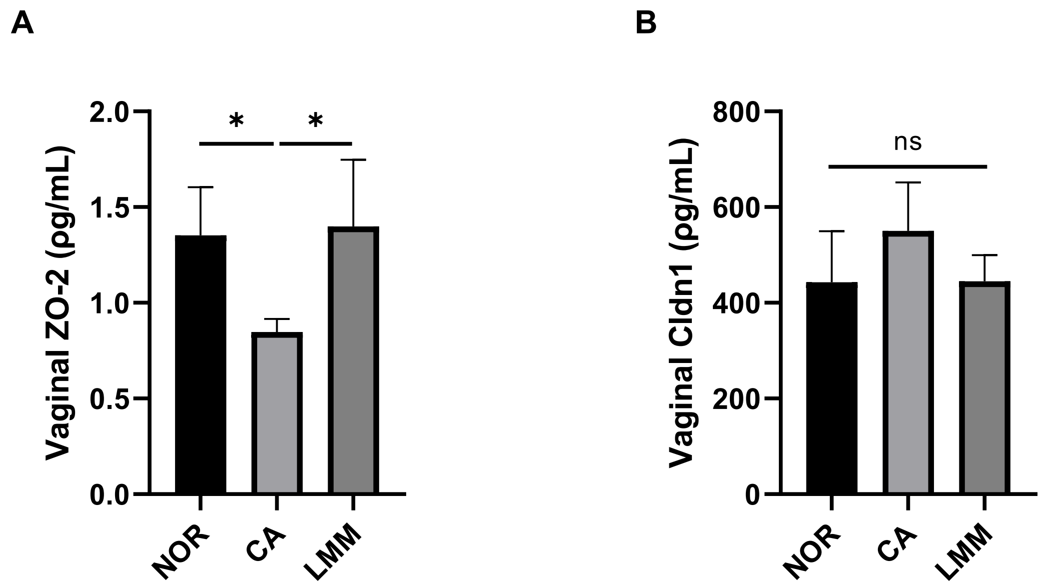

Fig. S2. Tight junction protein in the vaginal tissue from *C. albicans*-infected mice.
Data represent the mean ± SD (n=5).
Statistical significance was calculated using Prism 9. $^{*}p<0.05$.
NOR: Normal group
CA: *C. albicans* (1×10^{8} CFU/20ul) infected group
LMM: *C. albicans*-infected middle LM (2×10^{9} CFU) group

Table S6. NanoString mRNA expression levels of LM in vaginal tissue of *C. albicans*-infected VVC mice.

Name	Gene name	Pathway	Fold change				
			CA vs. NOR	LML vs. CA	LMM vs. CA	LMH vs. CA	UREX vs. CA
ALOX15	Arachidonate 15-Lipoxygenase	Inflammation, lipid metabolism	2.49†	-1.63*	-5.18*	-5.10*	-5.45*
PPARA	Peroxisome proliferator activated receptor alpha	Lipid metabolism	-3.37†	1.38	1.54	-1.49	3.99*
PPARG	Peroxisome proliferator activated receptor gamma	Lipid metabolism	5.77†	-3.51*	-2.54	-3.49*	-1.91
Adipoq	Adiponectin	Glucose and fatty acid oxidation	5.71†	-38.73*	-15.14*	-13.08*	-4.69*
Lep	Leptin	Energy homeostasis	32.72†	-177.12*	-72.64*	-154.99*	-75.68*
CD36	Cluster of differentiation 36	Fatty acid transport	5.49†	-7.57*	-7.08*	-9.21*	-5.40*
CPT1A	Carnitine palmitoyltransferase 1A	Fatty acid β-oxidation	-1.86†	1.03	-1.21	1.02	1.17
SCD1	Stearolyl-Coenzyme A desaturase 1	Fatty acid metabolism	2.44^{NS}	-2.06	-1.53	4.09	-1.82
SREBF1	Sterol regulatory element binding transcription factor 1	Sterol biosynthesis	1.40	1.30	2.10*	-1.05	-1.55
LPL	Lipoprotein lipase	Lipid metabolism	-1.82	1.26	3.48	5.91*	4.55
BAX	BCL2 associated X	Apotosis	2.13†	-4.45*	-2.36*	-2.13*	-2.08*
BCL2L1	BCL2 like 1	Apotosis	-1.65	1.60	1.89	1.26	3.33*
ATF2	Activating Transcription Factor2	Apotosis, inflammation	4.42†	-2.89*	-2.38*	-5.44*	-3.20*
CREB1	cAMP responsive element binding protein	Induction of transcription of genes	1.13^{NS}	-1.34	-2.08	-1.88	-1.33
CRP	C-reactive protein	Inflammation	-1.87^{NS}	-4.17	-1.23	-2.54	-1.72

CSF1	Colony stimulating factor 1	Inflammation	5.14†	-5.42*	-2.05*	-2.86*	-5.07*
CXCL12	C-X-C motif chemokine ligand 12	Inflammation	2.22NS	-1.77	-1.91	-1.50	-3.17
IL10	Interleukin 10	Inflammation	-1.34NS	-1.47	-1.02	-1.38	1.04
IL17A	Interleukin 17A	Inflammation	-1.08	9.14*	-1.90	-1.20	2.44
IL6	Interleukin 6	Inflammation	1.06 NS	-1.48	-1.09	-1.13	-1.31
IL22	Interleukin 22	Inflammation	-1.21NS	1.21	1.71	1.21	-1.18
JUN	Jun proto-oncogene, AP-1 transcription factor subunit	Inflammation	-1.27	-3.73*	-1.26	-1.02	1.10
KEAP1	Kelch like ECH associated protein 1	Nrf2 pathway	-1.34NS	-1.08	-1.47	-1.71	1.11
MAPK3	Mitogen-activated protein kinase 3	Proliferation, differentiation, cell cycle progression	1.04 NS	1.53	1.00	1.10	1.22
MMP-9	Matrix metallopeptidase 9	Inflammation	5.01†	-1.13	1.42	-1.82	2.76*
NFkB	Nuclear factor kappa B	Inflammation	1.01 NS	-1.09	-1.16	1.39	1.20
NFkBIA	NFKB inhibitor alpha	Inflammation	1.11 NS	1.06	-1.05	-1.07	1.01
NOS2	Nitric oxide synthase 2	Inflammation	10.32†	-16.81*	-14.45*	-12.99*	-13.34*
NOX1	NADPH oxidase 1	Oxidative stress response	2.66	-8.12*	-3.21	-7.48*	-3.55
PTGS2	Prostaglandin endoperoxide synthase 2	Inflammation	2.73 NS	1.15	-1.47	-2.42	-1.57
RAC1	Rac family small GTPase 1	MAPK signaling	1.61	-1.07	-1.12	-1.21	2.04*
RELA	NF-kB subunit	Inflammation	-1.49	1.56	2.11*	1.35	2.12
SMAD7	SMAD family member 7	Fibrogenesis	-2.94†	-1.02	2.93*	3.29*	2.90*
STAT3	Signal transducer and activator of transcription 3	Cell growth and apoptosis	1.32NS	1.05	-1.41	-1.65	-1.08

TGFB2	Transforming growth factor beta 2	Inflammation, fibrosis	-1.07 ^{NS}	-1.03	-1.10	-1.20	-1.05
TNF	Tumor necrosis factor	Inflammation	1.74 ^{NS}	-1.58	-1.32	1.54	1.04
XBP1	X-box binding protein 1	Unfolded protein response	1.52†	-1.04	-1.51	-1.63*	-1.08
GAPDH			1				
HPRT							
POLR1B							

Data represent the mean ± SD (n=5). Statistical significance was calculated using Prism 9. $^{*}p$<0.05, $^{**}p$<0.01, $^{***}p$<0.001, $^{****}p$<0.0001.

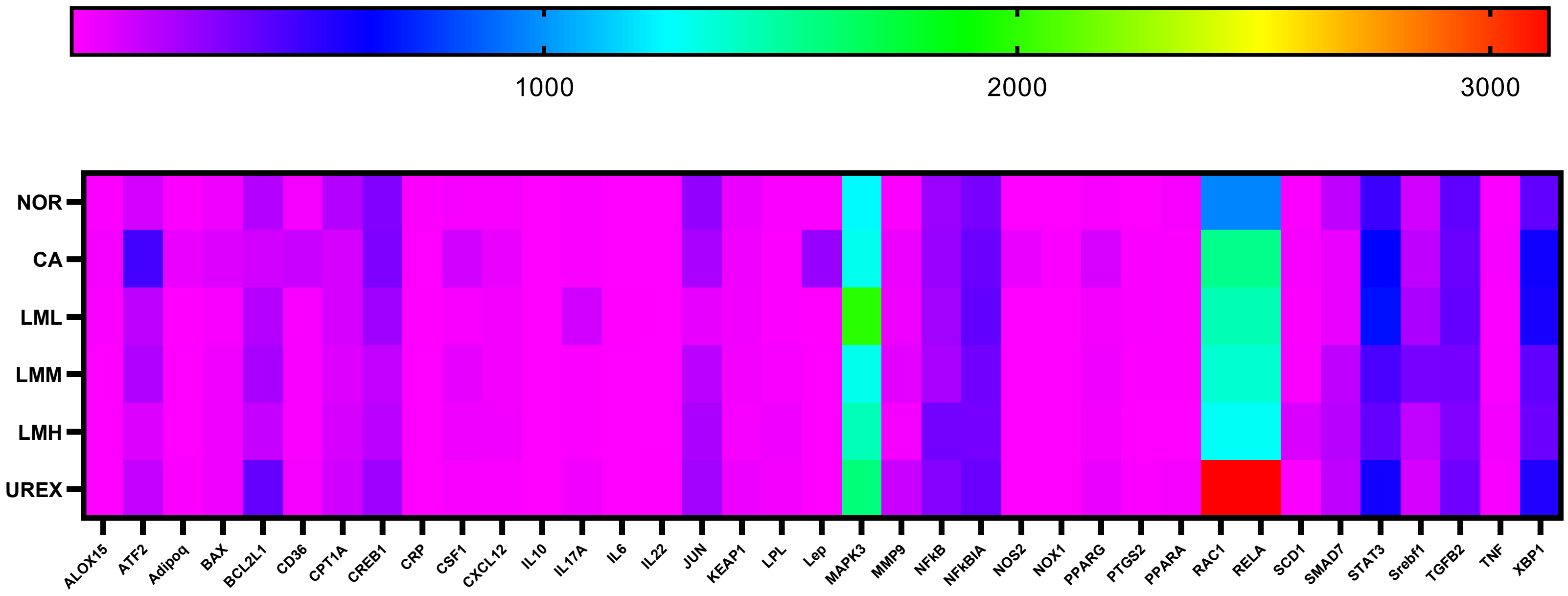

Fig. S2. **Heat map of NanoString gene expression in vaginal tissue**.
